# Anti-cytokine autoantibodies linked to susceptibility, bacterial load, and outcome in pneumococcal meningitis: prospective cohort studies in CNS infections, Alzheimer's disease, and Parkinson's disease

**DOI:** 10.1016/j.ebiom.2025.105975

**Published:** 2025-10-28

**Authors:** Rutger Koning, Nora Chekrouni, Lindsey B. Rosen, Marian A. van Roon, Anke A.G. Tolido, Evelien H.G.M. Drost, Dixie Bakker, Steven L. Staal, Sabine E. Olie, Wing Kit Man, Wilma D.J. van de Berg, Yolande A.L. Pijnenburg, Steven M. Holland, Matthijs C. Brouwer, Diederik van de Beek

**Affiliations:** a Department of Neurology, Amsterdam Neuroscience, Amsterdam UMC, University of Amsterdam, Amsterdam, the Netherlands; b Laboratory of Clinical Immunology and Microbiology, National Institute of Allergy and Infectious Diseases (NIAID), National Institutes of Health (NIH), Bethesda, MD, USA; c Department of Anatomy and Neurosciences, Section Clinical Neuroanatomy and Biobanking, Amsterdam Neuroscience, Amsterdam UMC, Vrije University Amsterdam, Amsterdam, the Netherlands; d Amsterdam Neuroscience, Program Neurodegeneration, Amsterdam, the Netherlands; e Alzheimer Centre Amsterdam, Neurology, Amsterdam Neuroscience, Amsterdam UMC, University of Amsterdam, Amsterdam, the Netherlands

**Keywords:** Bacterial meningitis, Anti-cytokine autoantibodies, *Streptococcus pneumoniae*, CNS infections, Meningitis, Encephalitis

## Abstract

**Background:**

*Streptococcus pneumoniae* is a nasopharyngeal coloniser but can also cause life-threatening invasive disease such as meningitis. The role of anti-cytokine autoantibodies as mechanism of acquired immunomodulation and immunodeficiency has recently been recognised.

**Methods:**

This study investigates the presence and potential role of anti-cytokine autoantibodies in central nervous system infections by analysing 14 anti-cytokine autoantibodies in the cerebrospinal fluid of 623 patients with pneumococcal meningitis, 86 with meningococcal meningitis, 56 with viral meningoencephalitis, 206 with Alzheimer's disease, 61 with Parkinson's disease, and 264 healthy controls from prospective cohort studies.

**Findings:**

Anti-cytokine autoantibodies were detected at significantly higher frequencies in patients with pneumococcal meningitis than in uninfected controls, particularly against IFN-γ (11% vs. 1%) and IFN-ω (11% vs. 0%), IL-1α (5% vs. 0%), IL-6 (12% vs. 1%), and IL-17A (9% vs. 1%) and IL-17F (10% vs. 0%), suggesting a role in disease susceptibility. In meningococcal meningitis, antibodies targeting IFN-γ (10%), IL-1α (7%), and IL-17F (7%) were associated with infection, while antibodies against IFN-γ were more prevalent in viral meningoencephalitis (12%). In Alzheimer's and Parkinson's disease, prevalence of autoantibodies was similar to controls. In pneumococcal meningitis, anti-IL-17A antibodies correlated with bacterial loads, and antibodies against IL-17A and IFN-ω were independently associated with death (adjusted odds ratios 3.06 [95% CI 1.35–6.92; p = 0.007]; 2.15 [95% CI 1.06–4.34; p = 0.033]). Only one of the autoantibodies against IFN-ω and none of the autoantibodies against IL-17A were shown to be neutralising.

**Interpretation:**

Anti-cytokine autoantibodies are present in the CSF of patients with central nervous system infections, particularly in pneumococcal meningitis. The observed correlation between these autoantibodies and death, as well as elevated bacterial loads in CSF, suggest that they might actively shape disease progression; however, further research is needed to establish a causal role.

**Funding:**

Nederlandse organisatie voor Wetenschappelijk Onderzoek, ItsME Foundation, 10.13039/501100000781European Research Council, Dutch Parkinson Foundation, Neuroscience Campus Amsterdam, ZonMW, Van Alkemade fonds, Stichting Woelse Waard, 10.13039/501100008358Hersenstichting and 10.13039/100006483AbbVie.

**Research in context**

**Evidence before this study**

A systematic search was performed in Pubmed on March 3rd 2025, to identify studies evaluating anti-cytokine autoantibodies in bacterial meningitis. Search terms related to “bacterial meningitis”, “antibodies” and “cytokines” were used. One small study by Takasaki et al., identified increased levels of autoantibodies targeting IL-8 in the cerebrospinal fluid of nine children with bacterial meningitis compared to twelve with aseptic meningitis and eight controls. Previous reports have linked the presence of anti-cytokine autoantibodies targeting type I interferons with susceptibility to West-Nile virus encephalitis, and antibodies against GM-CSF were found in patients with cryptococcal meningitis. However, the role of these autoantibodies in bacterial meningitis remains largely unexplored.

**Added value of this study**

In this study we investigated the presence of anti-cytokine autoantibodies in patients with pneumococcal and meningococcal meningitis, comparing them to patients with viral meningoencephalitis, Alzheimer's and Parkinson's disease, and controls. In central nervous system infections, autoantibodies were highly prevalent in the cerebrospinal fluid compared to controls. In pneumococcal meningitis, the presence of anti-cytokine autoantibodies was independently associated with increased mortality and higher bacterial loads in the CSF.

**Implications of all the available evidence**

Our findings show a possible pathogenic role of anti-cytokine autoantibodies in central nervous system infection, particularly pneumococcal meningitis, and their potential as biomarkers for risk stratification and therapeutic targets in this disease. Further research is necessary to determine if these autoantibodies causally influence disease progression.

## Introduction

Bacterial meningitis is a life-threatening disease, caused by bacterial invasion of the central nervous system.^1^ *Streptococcus pneumoniae* and *Neisseria meningitidis*, common colonisers of the nasopharynx, are the most frequent causative pathogens.^1^ Despite advances in treatment, notably adjunctive dexamethasone, and childhood vaccination programs, the burden of disease caused by bacterial meningitis remains high.^2^^,^^3^ Several clinical risk factors for unfavourable outcome and mortality have been identified, but the observed differences in disease susceptibility and disease course between patients are incompletely understood.^2^

Recent research has highlighted the role of anti-cytokine autoantibodies in modulating immune responses in infectious and inflammatory diseases.^4^ These autoantibodies, typically polyclonal IgG, can neutralise cytokines, disrupt their signalling pathways, and influence disease severity.^5^ Furthermore, it has been hypothesised that anti-cytokine autoantibodies can alter the half-life of cytokines in the circulation, and might act as carrier proteins for their target cytokine, thereby increasing bio-availability.^5^ In COVID-19, autoantibodies neutralising type I interferons (IFN-I) have been associated with severe disease and death.^6^ In central nervous system infections, elevated anti-IL-8 antibodies were reported in children with bacterial meningitis,^7^ anti-IFN-I antibodies detected in patients with West Nile encephalitis,^8^ and anti-GM-CSF antibodies associated with cryptococcal meningitis.^9^

We investigated the presence of anti-cytokine autoantibodies in CSF from patients with pneumococcal meningitis, meningococcal meningitis and viral meningitis or encephalitis. We also investigated the presence of these antibodies in patients with Alzheimer's and Parkinson's disease, as recent evidence highlights the role of the immune system in disease progression of these neurodegenerative disorders.^10^^,^^11^ In pneumococcal meningitis, we examined the association of autoantibodies with disease susceptibility, bacterial loads, and outcomes and analysed the dynamics of these antibodies in plasma.

## Methods

### Prospective cohorts of patients and controls

Patients with bacterial meningitis were selected from the MeninGene study, which is an ongoing prospective nationwide cohort study on community-acquired bacterial meningitis.^12^ In short, the study investigators are notified by the Netherlands Reference Laboratory of Bacterial Meningitis upon receipt of a positive CSF bacterial strain. Patients aged 16 years or older are eligible for inclusion. For the current study, CSF samples collected between 2006 and 2022 were selected if the patient had a positive CSF culture for *S. pneumoniae* or *N. meningitidis*. Patients with a negative CSF culture were included if they had a positive CSF polymerase chain reaction for these pathogens together with at least one individual CSF finding predictive of bacterial meningitis according to the Spanos criteria.^13^ CSF from the diagnostic puncture were centrifuged (10 min, 4500×*g*, at room temperature) and stored at −70 °C until further analysis. A subset of MeninGene patients was also included in the Serial Meningitis Sampling (SMS) substudy. Blood sampling is performed on day 0, 1, 2, 7 and 90, starting from the day of initial antibiotics administration. For the SMS study, blood samples collected between April 2015 and March 2022 were selected if patients met the same inclusion criteria as described above for the CSF samples. Blood samples were stored at −70 °C (Appendix p1).

Patients with PCR-proven viral meningitis and encephalitis were selected from 2017 to 2023 in the I-PACE study which has been described in detail previously (Appendix p2).^14^ For the Parkinson's disease cohort, we selected CSF samples from patients with Parkinson's from the Progress-PD and ‘Profiling Parkinson's (ProPark) cohorts, which have been described previously.^15^^,^^16^ For Progress-PD, patients with Parkinson's disease were recruited from the Amsterdam UMC, location VUmc outpatient clinic for movement disorders in the period 2008–2010 and 2017–2018 (Appendix p2). ProPARK is an ongoing longitudinal multicentre Parkinson's disease observational cohort study, for which individuals clinically diagnosed with Parkinson's disease and healthy controls were enrolled in the period 2021–2024 and annual follow-up is planned for three years (Appendix p2 and 3). For the Alzheimer's disease cohort, we selected CSF samples from the Amsterdam Dementia Cohort which was collected between March 2009 and August 2023 from the outpatient memory clinic (Appendix p2).^17^

For the healthy control cohort, we used CSF samples of healthy individuals included as controls in the Amsterdam Dementia Cohort and in the Parkinson Progress-PD cohort. Controls from the Amsterdam Dementia Cohort were individuals who were referred for cognitive complaints between November 2005 and July 2023, in whom all clinical investigations were normal (i.e. criteria for MCI or any psychiatric or neurological disorder not fulfilled; Appendix p3). For the Progress-PD, self-declared healthy controls were recruited through an advertisement in the periodical of the Dutch Parkinson Foundation, Dutch Brain Foundation (Hersenstichting), and via website of the Amsterdam UMC (Appendix p3).^15^ For the healthy plasma control cohort, we used plasma samples of healthy individuals included in the MeninGene Recall study (Appendix p3).^18^

### Detection of anti-cytokine autoantibodies

CSF and plasma samples from patients and controls were screened for autoantibodies using a multiplex particle-based assay (Bio-plex instrument, Bio-Rad; Appendix 4), as described before.^19^ In short, fourteen sets of magnetic beads were activated and covalently coupled to 2.5 μg recombinant human GM-CSF, IFN-α, IFN-β, IFN-γ, IFN-λ2, IFN-λ3, IFN-ω, IL-1α, IL-6, IL-12p70, IL-17A, IL-17F, IL-23 and TGF-β. For the initial pilot run, additional beads were coupled to complement component 3a (C3a), C5a, C-X-C motif chemokine ligand (CXCL) 10, IFN-λ1, IL-10, IL-15, IL-22, IL-33, macrophage colony-stimulating factor (M-CSF), tumour-necrosis factor (TNF)-α and TNF-β. The full panel was measured in a pilot cohort of 197 patients with pneumococcal meningitis and 12 healthy controls (appendix p12), all other samples (CSF and plasma of all cohorts) were only tested using the final 14 target panel. The final targets were chosen based on reactivity in the pilot, previous research and physiological interest. To correct for aspecific binding in CSF to uncoupled beads, one set of magnetic beads was activated but not coupled to recombinant protein. To assess repeatability of results and monitor the inter-assay variability, negative control plasma and negative control CSF from healthy individuals, and positive control plasmas from known auto-antibody carrying individuals were run in every experiment on every plate. Different batches of bead coupling were validated for the same controls. For the assay CSF and plasma samples were centrifuged for 10 min (4200×*g*) and incubated with the combined coupled beads for 30 min at room temperature on a plate shaker (30 μl of CSF undiluted, 30 μl of plasma in 1:100 dilution). After incubation the beads were washed, and samples were incubated with PE-labelled anti-human IgG (ThermoFisher) for 30 min and washed again before being run in a multiplex assay on the Bio-plex 200 (Bio-Rad) instrument.

To determine specificity of the autoantibodies with a competitive binding assay, samples were pre-incubated with their target cytokine before the detection of autoantibodies (Appendix p5).

The fluorescent intensity (FI) for all samples and for each target was obtained from the multiplex particle-based assay. For both the CSF and the plasma samples the threshold for positivity was calculated for each target separately. A CSF sample was considered positive for a certain target if; (1) the FI was higher than the 99th percentile value of the same target in the healthy controls; and (2) the FI was higher than 500; and (3) the FI to the coupled beads exceeded binding to the uncoupled beads by three times. A plasma sample was considered positive if it met the first two requirements, as binding to uncoupled beads was not measured in plasma.

Functional neutralisation assays were performed to demonstrate a patient sample's neutralising activity by assessing STAT phosphorylation in peripheral blood mononuclear cells by flow cytometry (for IFN-γ, IFN-ω and IL-12p70), or by quantifying secreted embryonic alkaline phosphatase (SEAP) in HEK-Blue cell lines (IL-17A, IL-17F; Appendix p5 and 6, 18).

### Bacterial load quantification and cytokine measurements in CSF

Bacterial loads were determined in the CSF of a subset of 111 patients with pneumococcal meningitis using real-time PCR as previously described (Appendix p7).^20^ Cytokine levels were measured in the CSF of a subset of 402 patients with pneumococcal meningitis using a Luminex assay (R&D technologies) according to manufacturer's instructions.

### Statistical analysis

Continuous data is expressed as median with interquartile range and compared using Mann–Whitney U. Associations between continuous variables were examined using the Spearman rank correlation coefficient. Categorical data was compared using Fisher's exact test. Multivariable binary logistic regression was performed to assess the prognostic value of positivity for anti-cytokine autoantibodies for mortality adjusted for predefined risk factors, providing odds ratios (ORs) and 95% confidence intervals. Both univariable and multivariable ORs corrected for all other variables in the model were estimated. The assumption of linearity between a continuous variable and the (log odds of the) outcome was assessed by visual inspection. Missing data (3.8% of total values) were imputed using multiple imputation, by combining 5 imputed datasets based on all available prognostic factors. All tests were 2-tailed and statistical significance was defined as p < 0.05, with Bonferroni correction for the number of autoantibodies tested. Statistical analyses were conducted using SPSS (version 28) and RStudio (version 4.3.2).

### Role of the funding source

The sponsors had no role in the study design, analysis, or decision to submit for publication. We were not paid to write this article by a pharmaceutical company or other agency. The authors were not precluded from accessing data in the study and they accept responsibility to submit for publication.

### Ethics

For all studies written informed consent was obtained from all patients or their legally authorised representatives. The MeninGene study was approved by the Medical Ethics Committee of the Amsterdam UMC, location AMC, Amsterdam, The Netherlands (number METC 2013_043). The I-PACE study was approved by the biobank ethics committee of the Amsterdam UMC, location AMC, Amsterdam, The Netherlands (number BTC AMC2014_290). The procedures of the Progress-PD and ProPARK study and biobank were approved by the Medical Ethics committee of the Amsterdam UMC (METC reference number Progress-PD 2017-517; ProPARK 2019-515). Amsterdam Dementia Biobank has been approved by Medical Ethics Committee of the Amsterdam UMC, location VUmc (number METC 2017.315).

## Results

**Fig. 1:**
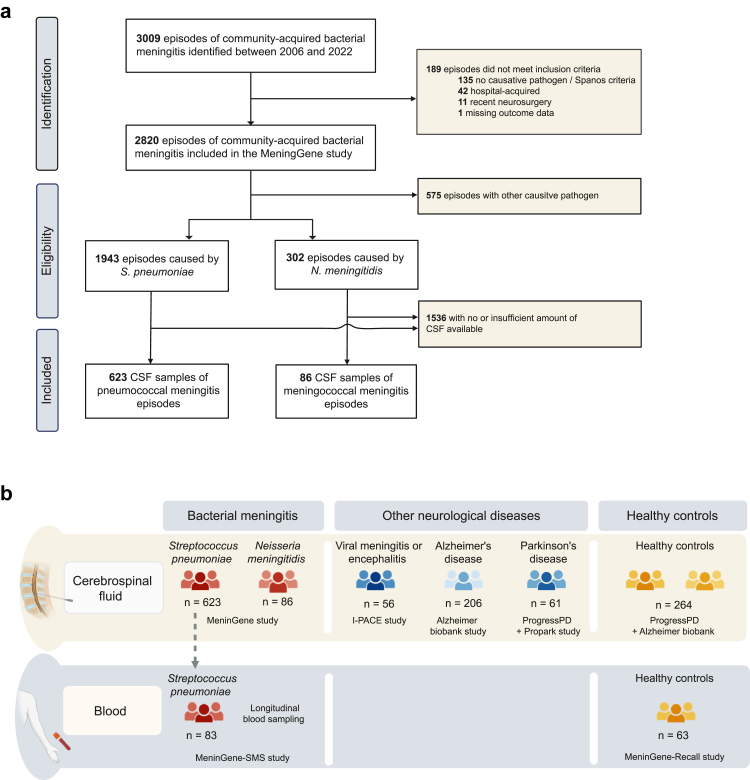
**Description of all cohorts. a**, Identification and inclusion of cerebrospinal fluid samples of pneumococcal and meningococcal meningitis patients from the MeninGene study. **b**, Overview of all cerebrospinal fluid and blood samples that were evaluated for the presence of autoantibodies against fourteen targets.

Between 2006 and July 2022, 2820 episodes of community-acquired bacterial meningitis were included in a prospective nationwide cohort study, including 1943 episodes caused by *S. pneumoniae* and 302 episodes by *N. meningitidis* (Fig. 1a).^12^ CSF from the diagnostic lumbar puncture was available from 623 pneumococcal and 86 meningococcal meningitis episodes. Baseline characteristics of patients with pneumococcal and meningococcal meningitis with and without CSF available were similar (Appendix p14). Additionally, we analysed CSF of 56 patients with viral meningitis or encephalitis, 206 patients with Alzheimer's disease, 61 with Parkinson's disease, and 264 healthy age-matched controls (Fig. 1b). Furthermore, 176 plasma samples of 83 patients with pneumococcal meningitis from different timepoints during disease were tested (Fig. 1b, Appendix p15).

The median age of patients with pneumococcal meningitis was 62 years, with 49% being female (Table 1). Symptoms were present less than 24 h in 49%, illustrating the acuteness of this severe illness. The classic triad of fever, neck stiffness, and altered mental status was present in 46% of episodes. On admission, 25% were comatose, and 27% had focal neurologic abnormalities. Cranial imaging revealed abnormalities in 51%, and adjunctive dexamethasone was administered in 94% of episodes. The median age of patients with meningococcal meningitis was 22 years and 52% were female (Appendix p16). For patients with viral meningitis or encephalitis the median age was 43 years and 63% of episodes occurred in females (Appendix p17).

**Table 1:** Baseline and clinical characteristics of pneumococcal meningitis cerebrospinal fluid cohort (n = 623).

Characteristic	Data	Characteristic	Data
Age, years^a^	62 (51–70)	**Indices of CSF inflammation**	
Female sex	307/624 (49%)	Protein (g ⁄ L)^e^	4.24 (2.63-6.28)
Symptoms <24 h	288/594 (49%)	CSF ⁄ blood glucose ratio^f^	0.04 (0.01–0.24)
Recurrent meningitis	40/622 (6%)	White cell count^g^	2755 (645–6667)
Extrameningeal focus of infection	340/618 (55%)	<100	65/615 (13%)
Otitis or sinusitis	286/604 (47%)	100–999	118/615 (23%)
Pneumonia or endocarditis	59/597 (12%)	1000–10000	329/615 (64%)
Endocarditis	11/602 (2%)	>10,000	103/615 (17%)
Immunocompromised state^b^	200/623 (32%)	**Microbiology blood and CSF**	
**Clinical signs and symptoms on admission**		Positive CSF culture	575/623 (92%)
Median temperature^c^	39.0 (38.0–39.7)	Positive blood culture	449/600 (75%)
Headache	425/525 (81%)	Only positive CSF PCR	18/623 (3%)
Neck stiffness	421/564 (75%)	**Glasgow Outcome Scale**	
Rash	19/532 (4%)	1 (death)	91/623 (15%)
Altered mental status (GCS <14)	498/622 (80%)	2 (vegetative state)	1/623 (0%)
Classic triad^d^	268/581 (46%)	3 (severe disability)	31/623 (5%)
Aphasia, monoparesis or hemiparesis	147/551 (27%)	4 (moderate disability)	113/623 (18%)
Seizures	61/598 (10%)	5 (mild or no disability)	387/623 (62%)

Data presented as n/N (%) or median (IQR). Group differences were tested with a Fisher's exact test for categorical variables and a Mann–Whitney U test for continuous variables. Abbreviations: CSF, cerebrospinal fluid; PCR, polymerase chain reaction.

a Age is known for all patients.

b Immunocompromised state is defined as active cancer (N = 89), diabetes (N = 103), alcoholism (N = 34), immunosuppressive treatment (N = 37), splenectomy (N = 21) or HIV (N = 5).

c Temperature is known for 608 episodes.

d Classic triad is defined as the combination of headache, neck stiffness and altered mental status.

e Protein level in CSF is known for 601 episodes.

f Glucose ratio is known for 580 episodes.

g White cell count in CSF is in cells per μl and is known for 615 episodes.

**Fig. 2:**
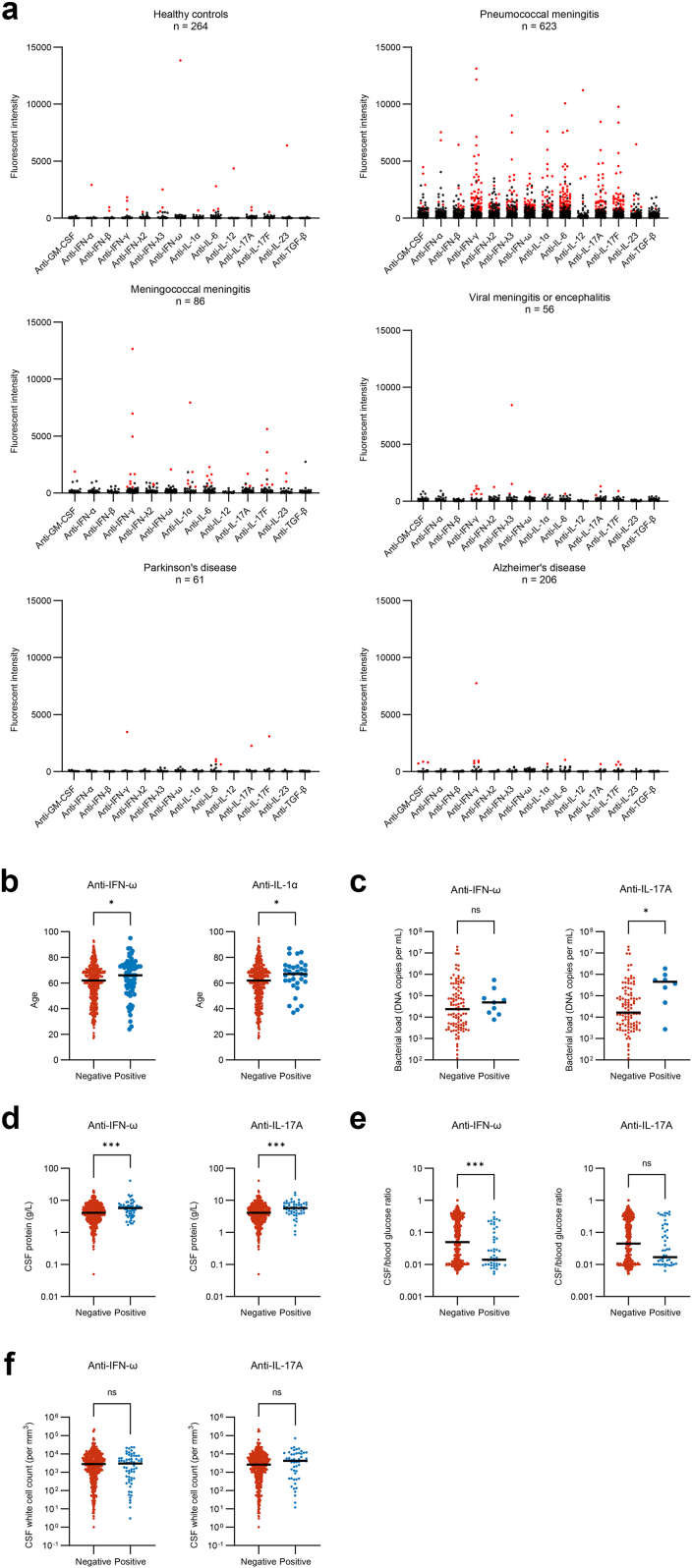
**Anti-cytokine autoantibodies in cerebrospinal fluid. a**, Multiplex particle-based assay for auto-antibodies against the 14 measured targets in cerebrospinal fluid of healthy controls (n = 264), pneumococcal meningitis (n = 623), meningococcal meningitis (n = 86), viral meningitis or encephalitis (n = 56), Parkinson's disease (n = 61) and Alzheimer's disease (n = 206). Samples that were considered positive (fluorescent intensity [FI] >99th percentile of the healthy cohort, >500 FI and >three times binding to uncoupled beads) are in red. **b**, Plot showing age (in years) between CSF samples positive and negative for autoantibodies against IFN-ω and IL-1α **c–f**, Plots showing bacterial load concentration (in DNA copies/mL) **(c)**, cerebrospinal fluid protein (in g/L) **(d)**, CSF/blood glucose ratio **(e)**, cerebrospinal fluid white cell count (in cells/mm^3^) **(f)**, in samples positive and negative for auto-antibodies against IL-17A or IFN-ω. Dark lines indicate the median values. Differences are calculated using the Mann–Whitney U test. ∗p value < 0.05; ∗∗p value < 0.01; ∗∗∗p value < 0.001.

For pneumococcal meningitis, 282 of 623 (45%) patients had at least one positive autoantibody against one of the measured targets, compared to 17 of 264 (6%) of healthy controls (Table 2; p < 0.001; Fig. 2a). Autoantibodies with a high prevalence in pneumococcal meningitis included anti-IFN-γ (66 of 623 [11%] vs. 3 of 264 [1%], p < 0.001), anti-IFN-λ3 (41 of 623 [7%] vs. 3 of 264 [1%], p = 0.01), anti-IFN-ω (65 of 623 [11%] vs. 1 of 264 [0%], p < 0.001), anti-IL-1α (32 of 623 [5%] vs. 1 of 264 [0%], p = 0.002), anti-IL-6 (75 of 623 [12%] vs. 3 of 264 [1%], p < 0.001), anti-IL-17A (55 of 623 [9%] vs. 2 of 264 [1%], p < 0.001), and anti-IL-17F (64 of 623 [10%] vs. 1 of 264 [0%], p < 0.001). A heatmap with clustering of the autoantibodies is shown in the supplementary data (Appendix p8). Advanced age was associated with the presence of autoantibodies against IFN-ω (66 vs. 62 years, p = 0.02) and IL-1α (67 vs. 62 years, p = 0.03) among patients with pneumococcal meningitis (Fig. 2b). There was no difference in occurrence of autoantibodies in patients that had symptoms on admission for longer vs. shorter than 24 h (respectively 140 of 306 [46%] vs. 125 of 288 [43%]; Fisher's exact p = 0.29).

**Table 2:** Presence of anti-cytokine autoantibodies in cerebrospinal fluid per cohort compared to healthy controls.

Antibody	Healthy controls (n = 264)	Pneumococcal meningitis (n = 623)	Meningococcal meningitis (n = 86)	Viral meningitis/encephalitis (n = 56)	Parkinson's disease (n = 61)	Alzheimer's disease (n = 206)
≥1 target positive	17/264 (6%)	282/623 (45%)∗∗∗	34/86 (40%)∗∗∗	13/56 (23%)∗∗	5/61 (8%)	13/206 (6%)
Anti-GM-CSF	0/264 (0%)	10/623 (2%)	1/86 (1%)	0/56 (0%)	0/61 (0%)	3/206 (1%)
Anti-IFN-α	1/264 (0%)	5/623 (1%)	0/86 (0%)	0/56 (0%)	0/61 (0%)	0/206 (0%)
Anti-IFN-β	2/264 (1%)	11/623 (2%)	0/86 (0%)	0/56 (0%)	0/61 (0%)	0/206 (0%)
Anti-IFN-γ	3/264 (1%)	66/623 (11%)∗∗∗	9/86 (11%)∗∗	7/56 (12%)∗∗	1/61 (2%)	5/206 (2%)
Anti-IFN-λ2	1/264 (0%)	18/623 (3%)	2/86 (2%)	1/56 (2%)	0/61 (0%)	0/206 (0%)
Anti-IFN-λ3	3/264 (1%)	41/623 (7%)∗∗	NA	3/56 (5%)	0/61 (0%)	0/206 (0%)
Anti-IFN-ω	1/264 (0%)	65/623 (10%)∗∗∗	1/86 (1%)	1/56 (2%)	0/61 (0%)	0/206 (0%)
Anti-IL-1α	1/264 (0%)	32/623 (5%)∗∗∗	6/86 (7%)∗	1/56 (2%)	0/61 (0%)	1/206 (0%)
Anti-IL-6	3/264 (1%)	75/623 (12%)∗∗∗	6/86 (7%)	1/56 (2%)	3/61 (5%)	1/206 (0%)
Anti-IL-12	1/264 (0%)	4/623 (1%)	0/86 (0%)	0/56 (0%)	0/61 (0%)	0/206 (0%)
Anti-IL-17A	2/264 (1%)	55/623 (9%)∗∗∗	3/86 (3%)	2/56 (4%)	1/61 (2%)	1/206 (0%)
Anti-IL-17F	1/264 (0%)	64/623 (10%)∗∗∗	6/86 (7%)∗	1/56 (2%)	1/61 (2%)	3/206 (1%)
Anti-IL-23	1/264 (0%)	8/623 (1%)	2/86 (2%)	0/56 (0%)	0/61 (0%)	0/206 (0%)
Anti-TGF-β	0/264 (0%)	1/623 (0%)	0/86 (0%)	0/56 (0%)	0/61 (0%)	0/206 (0%)

Data presented as n/N (%). Group differences between the healthy controls and each of the other cohorts were tested with a Fisher's exact test (Bonferroni corrected). ∗p-value <0.05. ∗∗p-value <0.01. ∗∗∗p-value <0.001.

Patients with meningococcal meningitis had a higher prevalence of anti-IFN-γ (9 of 86 [10%] vs. 3 of 264 [1%], p = 0.004), anti-IL-1α (6 of 86 [7%] vs. 1 of 264 [0%], p = 0.015), and anti-IL-17F (6 of 86 [7%] vs. 1 of 264 [0%], p = 0.015) compared to controls (Table 2). Viral meningoencephalitis also showed increased anti-IFN-γ autoantibodies (7 of 56 [12%] vs. 3 of 264 [1%]; p = 0.004), in patients with varicella zoster virus infection (five patients) and herpes simplex virus type 2 (two patients). No difference in the prevalence of CSF autoantibodies was found between patients with Alzheimer's and Parkinson's diseases compared to healthy controls.

Neutralisation assays were performed. For IL-1α and IL-6, cellular assays were stimulated by patients’ CSF independent of the presence of cytokines, rendering them uninterpretable. For IFN-ω, IFN-γ, IL-17A, and IL-17F, only two of 83 samples tested were neutralising (Appendix p18 and 19), potentially due to low antibody concentrations in the CSF.

The case fatality rate among patients with pneumococcal meningitis was 15%. Autoantibodies against IFN-ω, IL-1α, IL-17A and IL-17F were associated with death in univariate analysis (Table 3). In multivariate analysis, including known risk factors for death (age, immunocompromised state, heart rate on admission, Glasgow Coma Scale score on admission, cranial nerve palsies on admission, seizures on admission, C-reactive protein in blood and leucocyte count in CSF),^21^ the predictive effect of autoantibodies against IFN-ω and IL-17A on death remained robust (adjusted OR 2.15 [95% CI 1.06–4.34; p = 0.033] and 3.06 [95% CI 1.35–6.92; p = 0.007]).

**Table 3:** Univariable and multivariable logistic regression of anti-cytokine autoantibodies for mortality in the CSF pneumococcal meningitis cohort (N = 623).

	Survivors (N = 532)	Non-survivors (N = 91)	Univariable OR mortality (95% CI)	Multivariable OR mortality (95% CI)	Multivariable p-value
Presence of autoantibodies					
Anti-IFN-γ	55/532 (10%)	11/91 (12%)	1.19 (0.59–2.37)	–	–
Anti-IFN-λ3	34/532 (6%)	7/91 (8%)	1.22 (0.52–2.84)	–	–
Anti-IFN-ω	45/532 (9%)	20/91 (22%)	3.05 (1.70–5.46)^h^	2.15 (1.06–4.34)	0.033
Anti-IL-1α	23/532 (4%)	9/91 (10%)	2.43 (1.09–5.43)^f^	1.71 (0.62–4.70)	0.33
Anti-IL-6	59/532 (11%)	16/91 (18%)	1.71 (0.94–3.13)	–	–
Anti-IL-17A	39/532 (7%)	16/91 (18%)	2.69 (1.44–5.07)^g^	3.06 (1.35–6.92)	0.007
Anti-IL-17F	48/532 (9%)	16/91 (18%)	2.15 (1.16–3.98)^g^	1.70 (0.79–3.63)	0.16
Age, years^a^	62 (50–69)	69 (56–77)	1.14 (1.01–1.06)^h^	1.04 (1.01–1.06)	0.002
Female sex	262/532 (49%)	45/91 (49%)	1.00 (0.65–1.57)	–	–
Immunocompromised	151/532 (28%)	49/91 (54%)	2.84 (1.81–4.47)^h^	2.73 (1.58–4.71)	<0.001
Heart rate^b^	100 (86–112)	110 (95–130)	1.25 (1.14–1.38)^h^	1.14 (1.01–1.28)	0.033
GCS score^c^	11 (9–13)	9 (7–11)	0.84 (0.78–0.90)^h^	0.86 (0.79–0.95)	0.002
Cranial nerve palsy	33/456 (7%)	10/69 (14%)	2.25 (1.05–4.81)^f^	1.28 (0.51–3.19)	0.6
Seizures on admission	44/517 (9%)	17/81 (21%)	2.68 (1.43–5.04)^h^	2.52 (1.14–5.53)	0.022
C-reactive protein^d^	159 (80–278)	295 (202–396)	1.06 (1.04–1.07)^h^	1.05 (1.03–1.08)	<0.001
CSF white cell count^e^	3116 (976–7407)	526 (59–3413)	–	–	–
<100	42/526 (8%)	23/89 (26%)	4.96 (2.63–9.35)^h^	2.70 (1.19–6.13)	0.018
100–999	91/526 (17%)	27/89 (30%)	2.69 (1.53–4.71)^h^	3.16 (1.56–6.40)	0.001
1000–10,000	297/526 (56%)	32/89 (36%)	*Reference*	*Reference*	–
>10,000	96/526 (18%)	7/89 (8%)	0.74 (0.31–1.72)	0.79 (0.31–2.03)	0.63

Data presented as n/N (%) or median (IQR). Abbreviations: CSF, cerebrospinal fluid; GCS, Glasgow Coma Score; OR, odds ratio; 95% CI, 95% confidence interval.

a Age is known for all patients.

b Heart rate is shown per 10 beats per minute, and is known for 510 survivors and 86 non-survivors.

c Glasgow coma scale score is known for 532 survivors and 90 non-survivors.

d CRP is shown per 10 mg/L, and is known for 523 survivors and 85 non-survivors.

e White cell count in CSF is in cells per μl, and is known for 526 survivors and 89 non-survivors.

f Univariable odds ratio p-value <0.05.

g Univariable odds ratio p-value <0.01.

h Univariable odds ratio p-value <0.001.

CSF bacterial loads were determined in a subset of 111 patients with pneumococcal meningitis.^20^ Presence of IL-17A autoantibodies was associated with CSF bacterial load (median 4.6 × 10^5^ DNA copies/mL vs. 1.7 × 10^4^ [fold change = 27], p = 0.01; Fig. 2c). We also measured CSF IL-1β, TNF-α, IL-10 and IL-17A concentrations in 402 patients with pneumococcal meningitis (Appendix p9). Autoantibodies against IL-17A and IFN-ω were not associated with cytokine concentration after correction for multiple testing. Antibodies against IFN-ω were associated with CSF protein level (median 5.8 g/L vs. 4.1 g/L, p < 0.001, Fig. 2d) and CSF/blood glucose ratio (median 0.01 vs. 0.05, p < 0.001, Fig. 2e). These autoantibodies were not associated with CSF white cell count (Fig. 2f).

Paired blood and CSF samples were collected from 56 patients with pneumococcal meningitis. Among patients with anti-IFN-ω, 2 of 12 (17%) who tested positive in CSF also tested positive in blood, with one additional patient positive in blood only (Appendix p10). For anti-IL-17A, 4 of 5 (80%) patients positive in CSF tested positive in blood, highlighting a partial overlap between autoantibody detection in blood and CSF, with varying sensitivity depending on the cytokine target. Plasma samples were analysed from three distinct time points: the acute phase (days 0–2 after diagnosis) for all 83 patients (100%), the subacute phase (day 7) for 58 patients (70%), and the late phase (day 90) for 35 patients (42%; Table 4, Appendix p10). Among patients with anti-IFN-ω antibodies during the acute phase, four of five patients for whom later phase samples were available remained positive (Appendix p10). In contrast, among those without anti-IFN-ω antibodies in the acute phase, four developed positivity in the subacute phase and one in the late phase. For anti-IL-17A antibodies, two of the four patients who were positive during the acute phase remained positive in subsequent phases.

**Table 4:** Presence of anti-cytokine autoantibodies in blood per cohort compared to healthy controls.

Antibody	Healthy controls	Patients with pneumococcal meningitis		
		Acute (0–2 days)	7 days	90 days
Anti-GM-CSF	1/63 (2%)	0/83 (0%)	0/58 (0%)	0/35 (0%)
Anti-IFN-α	0/63 (0%)	1/83 (1%)	1/58 (2%)	0/35 (0%)
Anti-IFN-β	0/63 (0%)	8/83 (10%)	1/58 (2%)	0/35 (0%)
Anti-IFN-γ	1/63 (2%)	4/83 (5%)	5/58 (9%)	3/35 (9%)
Anti-IFN-λ2	1/63 (2%)	2/83 (2%)	0/58 (0%)	0/35 (0%)
Anti-IFN-λ3	1/63 (2%)	1/83 (1%)	1/58 (2%)	2/35 (6%)
Anti-IFN-ω	1/63 (2%)	5/83 (6%)	6/58 (10%)	3/35 (9%)
Anti-IL-1α	1/63 (2%)	6/83 (7%)	2/58 (3%)	2/35 (6%)
Anti-IL-6	1/63 (2%)	4/83 (5%)	12/58 (21%)∗∗	9/35 (26%)∗∗
Anti-IL-12	0/63 (0%)	7/83 (8%)	1/58 (2%)	0/35 (0%)
Anti-IL-17A	1/63 (2%)	4/83 (5%)	1/58 (2%)	1/35 (3%)
Anti-IL-17F	1/63 (2%)	8/83 (10%)	5/58 (9%)	6/35 (17%)
Anti-IL-23	1/63 (2%)	2/83 (2%)	1/58 (2%)	0/35 (0%)
Anti-TGF-β	0/63 (0%)	3/83 (4%)	2/58 (3%)	2/35 (6%)

Data presented as n/N (%). Group differences between the healthy controls and each of the other cohorts were tested with a Fisher's exact test (Bonferroni corrected). ∗p-value <0.05. ∗∗p-value <0.01.

## Discussion

We found a robust presence of anti-cytokine autoantibodies in various central nervous system infections. Anti-cytokine autoantibodies were highly prevalent in the CSF of patients with pneumococcal meningitis, meningococcal meningitis, and viral meningoencephalitis. For pneumococcal meningitis, we identified an association between these autoantibodies and outcome, with anti-IL-17A and anti-IFN-ω emerging as independent predictors of mortality. The observed correlation between these autoantibodies and elevated bacterial loads in CSF, as well as their persistence in blood, suggest that they might play an active role in disease progression, although further studies are needed to explore whether these autoantibodies are causally linked to disease course. Insights into the functional effects of these autoantibodies–whether through neutralisation or modulation of cytokine signalling, highlight their potential as biomarkers for risk stratification and as novel therapeutic targets to improve patient outcomes.

While inborn errors of the type I interferon (IFN) pathway have been primarily associated with severe viral infections,^22^ and genetic deficiencies in the IL-17 pathway are classically linked to chronic mucocutaneous candidiasis and other opportunistic infections, emerging evidence indicates that patients with these defects may also exhibit an increased susceptibility to bacterial infections, including those caused by encapsulated pathogens such as *S. pneumoniae*.^23^ Unlike congenital genetic deficiencies, anti-cytokine autoantibodies are acquired later in life and therefore exhibit distinct kinetics and tissue distribution. It is worth noting that patients in our cohort were not selected based on underlying inborn errors of immunity but represent an unselected clinical cohort of severe CNS infections. An important question is whether these autoantibodies could be induced by the infection itself, or whether their presence represents a predisposing factor for disease development. In our study, CSF samples were collected at the time of diagnostic lumbar puncture, either prior to or shortly after initiation of antimicrobial therapy. Notably, 49% of patients with pneumococcal meningitis had experienced symptoms for less than 24 h at the time of presentation, illustrating the acute onset of the disease. This makes it highly unlikely that the autoantibodies were generated during the early stages of infection. Instead, our findings suggest that these autoantibodies were already present prior to disease onset and may contribute to disease progression. Supporting this, the proportion of patients with at least one detectable autoantibody did not differ between those with symptom duration shorter or longer than 24 h.

IL-17A and IL-17F, which share a receptor complex (IL-17RA/IL-17RC), play a critical role in immunity against extracellular pathogens. Autoantibodies against both cytokines were associated with mortality in our pneumococcal meningitis cohort, with IL-17A showing a stronger effect in a multivariate analysis. The discovery that IL-17A autoantibodies correlate with increased bacterial loads aligns with existing knowledge about IL-17A's role in regulating host defences against extracellular pathogens.^24^ Recent studies showed that IL-17 signalling plays a key role in the host defence against pneumococcal infections, including pneumococcal meningitis.^25^ Genetic variation in IL-17 was shown to be associated with both increased susceptibility and severity of pneumococcal disease.^25^ Anti-IL-17 autoantibodies might reduce the ability of IL-17 to prevent pneumococcal colonisation of the nasopharynx.^26^ A disturbed regulation of IL-17 mediated leucocyte chemotaxis and pneumococcal killing might well explain the observed enhanced disease severity.^24^

The use of secukinumab, a therapeutic monoclonal antibody targeting IL-17A, may offer insights into the potential effects of IL-17A autoantibodies in the CSF. While secukinumab is associated with increased risk of minor and opportunistic infections, reports of serious infections or central nervous system involvement are rare.^27^ To date, only a single case report has described streptococcal brain abscesses in a patient receiving secukinumab, suggesting this may represent an isolated event.^28^ It is important to note that secukinumab is a strong neutraliser of IL-17 signalling, while in our study none of the CSF samples positive for IL-17A autoantibodies exhibited neutralising activity. Therefore these endogenous antibodies might exert different effects than therapeutic blockade by secukinumab.

Antibodies against IFN-ω were also associated with death. Autoantibodies against type I interferons, including anti-IFN-ω, have previously been associated with severity of viral infections including COVID-19 pneumonia and West Nile virus disease.^29^ However, their role in invasive pneumococcal disease has not been reported. Similar to findings in viral infections, the observed prevalence of autoantibodies increased with age.^6^ The importance of type I interferons in pneumococcal meningitis was highlighted in a study showing that increased activation of the type I interferon pathway correlates with higher mortality.^30^ In West Nile virus infection, it was suggested that autoantibodies targeting type I interferons compromise the blood–brain barrier, thereby increasing susceptibility to encephalitis.^8^ A similar mechanism may well be relevant in the pathophysiology of pneumococcal meningitis. Interestingly, we did not find a correlation between the presence of autoantibodies against type I interferons in our viral meningitis or encephalitis cohort. This might be mostly due to the heterogeneity in causative pathogens together with the relatively limited number of samples.

We observed a correlation between the presence of autoantibodies in both CSF and plasma, suggesting that the production of autoantibodies is part of a systemic response. However, in some patients, specific autoantibodies were detected exclusively in one compartment, indicating a localised autoantibody response in these patients. The dynamics of autoantibodies across the acute, subacute, and late phases of disease, suggest that these antibodies can have a durable and transient immune modulation. Unfortunately, only a limited number of paired CSF-plasma samples from patients with pneumococcal meningitis were available for the measurements, and only some of these had positive autoantibodies in CSF. Future research should investigate whether these autoantibodies are induced by bacterial antigens, genetic predisposition, or environmental influences, offering further insight into their role in disease pathogenesis and potential therapeutic opportunities.

In patients with Alzheimer's and Parkinson's disease, anti-cytokine antibodies were no more prevalent in CSF than in healthy controls. While these are primarily viewed as neurodegenerative diseases, growing evidence suggests that the immune system plays a role in their development and progression.^31^ The potential involvement of anti-cytokine autoantibodies in these diseases has not been previously investigated, although autoantibodies targeting other antigens have been documented.^32^ The absence of detectable anti-cytokine autoantibodies in the CSF of patients with Alzheimer's and Parkinson's disease suggests that these autoantibodies are unlikely to play a major role in the pathophysiology of these diseases.

Our study has limitations. First, only patients without contraindications to lumbar puncture, such as space-occupying lesions on cranial CT or septic shock, were included, omitting a subset of critically ill patients, potentially underestimating both the mortality and the impact of autoantibodies on outcome.

Second, to address potential confounding by disease severity, we performed a multivariate logistic regression analysis to evaluate the relationship between anti-cytokine antibodies and outcome. We recognise that multicollinearity can occur when independent variables are highly correlated; however, as our aim was to use logistic regression for outcome prediction (classification into favourable vs. unfavourable outcome), any multicollinearity present does not distort the predictive performance of the model.

Third, we were unable to fully determine the functional role of the autoantibodies. This limitation stemmed partly from the reactive nature of the CSF from patients with central nervous system infections, with a subset of CSF samples activating cellular-based assays even in absence of cytokines, preventing the evaluation of neutralising capacity of some autoantibodies. Among the autoantibodies that we could test, including those targeting IFN-ω and IL-17A, most did not neutralise. It remains possible that these autoantibodies are capable of neutralising cytokine levels lower than those tested. Nonetheless, we should consider the possibility that these autoantibodies do not possess neutralising capacity, as has been described previously in literature.^33^ The exact functional implications of non-neutralising anti-cytokine autoantibodies detected in the CSF remain unclear; however, several potential roles can be considered. One potential role is to prolong the half-life of cytokines by protecting them against proteolytic degradation, as described for autoantibodies against IL-6 and IFN-γ.^33^^,^^34^ Prolonged cytokine availability could, in turn, modulate the dynamics of CNS inflammation. Alternatively, these autoantibodies may represent a bystander phenomenon arising from a dysregulated immune environment within the CNS, where there is abnormal antigen exposure and loss of immune tolerance. In this case, these autoantibodies would not have direct functional consequences.

Fourth, polyreactivity in a considerable proportion of samples necessitated a conservative approach to define positivity. This may have led to an underestimation of the prevalence of anti-cytokine antibodies in our cohort. Nevertheless, we were able to correlate our findings with severity and outcome, strengthening the likely importance of our findings.

Lastly, while multiplexed assays present inherent challenges—such as potential issues with sensitivity, specificity, and cross-reactivity—the comparability of results with established single-analyte assays is crucial. In our study, control samples were analysed using both multiplex and single-analyte platforms, yielding comparable results. This supports the reliability of the multiplexed approach in detecting autoantibodies.

Our findings show that levels of anti-cytokine autoantibodies are increased in CSF during pneumococcal meningitis. The association of antibodies against IFN-ω and IL-17A with mortality suggests that these antibodies can actively shape disease progression and outcome. We suggest potential value in screening for autoantibodies to identify patients who might receive extra benefit from pneumococcal vaccination. Future studies should address the timing at which these autoantibodies appear and their functional role. To this end, patient studies should be used, especially those measuring the presence of autoantibodies in the blood and CSF at different time points. Additionally experimental models, including mouse models for bacterial meningitis^35^^,^^36^ or in *ex vivo* organotypic brain slice cultures^37^^,^^38^ could help elucidate the functional role of these antibodies.

## Contributors

All authors read and approved the final version of the manuscript.

Rutger Koning: data curation, formal analysis, investigation, methodology, validation, visualisation, writing—original draft.

Nora Chekrouni: data curation, formal analysis, investigation, methodology, validation, visualisation, writing—original draft.

Lindsey B. Rosen: methodology, writing—review & editing, validation.

Marian A. van Roon: investigation, validation, writing—review & editing.

Anke A.G. Tolido: investigation, methodology, writing—review & editing.

Evelien H.G.M. Drost: investigation.

Dixie Bakker: investigation, writing—review & editing.

Steven S. Staal: investigation, writing—review & editing.

Sabine E. Olie: investigation.

Wing Kit Man: investigation.

Wilma D.J. van de Berg: funding acquisition, resources, writing—review & editing.

Yolande A.L. Pijnenburg: resources, writing—review & editing.

Steven M. Holland: resources, writing—review & editing.

Matthijs C. Brouwer: conceptualisation, project administration, resources, supervision, writing—review & editing.

Diederik van de Beek: conceptualisation, funding acquisition, project administration, resources, supervision, writing—review & editing.

## Data sharing statement

The data generated and analysed in this study are available from the corresponding author upon reasonable request. Due to patient confidentiality and ethical restrictions, access to the data may require institutional review and approval.

## Declaration of interests

WvdB has performed contract research for Discoveric Bio, AC Immune, Nitrase Therapeutics, La Hoffmann-La Roche and Gain Therapeutics. She is a member of the scientific advisory board of Gain Therapeutics.
