# Supplementary material for: Anti-cytokine autoantibodies linked to susceptibility, bacterial load, and outcome in pneumococcal meningitis: prospective cohort studies in CNS infections, Alzheimer's disease, and Parkinson's disease
Source: eBioMedicine. 2025 Oct 28;121:105975. doi: 10.1016/j.ebiom.2025.105975 (PMC12597061; doi:10.1016/j.ebiom.2025.105975)
Supplement: Supplementary Data [file mmc1.pdf]

SUPPLEMENTARY MATERIAL

Anti-cytokine autoantibodies linked to susceptibility, bacterial load, and outcome in pneumococcal meningitis: prospective cohort studies in CNS infections, Alzheimer's disease, and Parkinson's disease

SUPPLEMENTARY METHODS

p1. Bacterial meningitis cohort

p2. Other neurological diseases control cohorts

p3. Healthy control cohort

p4. Reagents list

p5. Competitive binding assay

p6. Functional evaluation of anti-cytokine autoantibodies

p7. Bacterial load quantification and cytokine measurements in CSF

SUPPLEMENTARY FIGURES

p8. Suppl. Figure 1. Heatmap with positive autoantibody in CSG of pneumococcal meningitis patients

p9. Suppl. Figure 2. Cytokine measurements in the CSF of pneumococcal meningitis patients

p10. Suppl. Figure 3. Autoantibody measurements in the plasma of pneumococcal meningitis patients

p11. Suppl. Figure 4. Polyreactivity

p12. Suppl. Figure 5. Pilot anticytokine autoantibody measurements

p13. Suppl. Figure 6. Representative neutralisation assays

SUPPLEMENTARY TABLES

p14. Suppl. Table 1. Baseline and clinical characteristics comparing pneumococcal and meningococcal meningitis patients included in the MeninGene study with and without cerebrospinal fluid available

p15. Suppl. Table 2. Baseline and clinical characteristics of plasma cohort *S. pneumoniae* meningitis

p16. Suppl. Table 3. Baseline and clinical characteristics of CSF cohort of *N. meningitidis* meningitis

p17. Suppl. Table 4. Baseline characteristics of non-bacterial meningitis cohorts

p18. Suppl. Table 5. Neutralising capacity of cerebrospinal fluid or blood of bacterial meningitis patients

p19. Suppl. Table 6. Functional assays to assess biological activity of anticytokine autoantibodies *in vitro*

SUPPLEMENTARY REFERENCES

SUPPLEMENTARY METHODS

p1. Bacterial meningitis cohort

Sample selection and inclusion

For patients included in the SMS study, blood samples were used if at least 10 µl was available, and if patients met the same inclusion criteria as described for the CSF samples. For each time point, plasma (in EDTA tubes) and serum (in clot tubes) samples were collected. Plasma was centrifuged (10 minutes, 2000 x g, at room temperature) and stored at -70 °C. The serum tubes were allowed to clot for 60 minutes, and subsequently centrifuged (10 minutes, 2000 x g, at room temperature), after which the supernatant was stored at -70 °C.

Data collection and definitions

Patient data was collected using a case record form. Patients were immunocompromised if they were using immunosuppressive drugs or had a medical history of diabetes mellitus, active cancer, alcoholism, human immunodeficiency virus (HIV) infection or splenectomy. Outcome was assessed at discharge using the Glasgow Outcome Scale (GOS) score, ranging from a score of 1=death, to a score of 5=mild or no disability.

p2. Other neurological diseases control cohorts

I-PACE

The I-PACE study is an ongoing multicentre cohort study in the Netherlands on adults with a clinical suspicion of a CNS infection who underwent CSF examination.[1] Exclusion criteria for this study were recent head trauma (≤1 month), recent neurosurgery (≤1 month) or a neurosurgical device in situ. CSF samples from the diagnostic lumbar puncture were selected if at least 50 µl was available. CSF was centrifuged and supernatant was stored at -70 °C until analysis.

Progress-PD and ProPARK

For the Progress-PD study, CSF was selected from the Progress-PD biobank of Parkinson's disease patients with a maximum 5 years of disease duration and Parkinson's disease patients with a Mini-Mental State Examination (MMSE) score > 19 (n=42). For the ProPARK study, CSF was collected at baseline from a subset of Parkinson's disease patients at the Amsterdam UMC Parkinson and Movement Disorders centre in the period 2021-2022 (n=19) and stored in the ProPARK biobank. Parkinson's disease patients were included if they fulfilled the clinical diagnostic criteria of the Movement Disorder Society and United Kingdom Parkinson's Disease Society

Brain Bank (UK-PDSBB) clinical diagnostic criteria, aged ≥ 18 year and maximum disease duration of ≤15 years.[2,3] They were excluded if on advanced therapies (i.e. levodopa continuous intestinal gel, apomorphine treatment or deep brain stimulation) or Montreal Cognitive assessment (MoCA) scale of ≤16. CSF was obtained by lumbar puncture, collected in polypropylene collection tubes, centrifuged at 1800 × g at 4 °C for 10 min, aliquoted and stored at −80 °C within 2 h in the central biobank of Amsterdam UMC, according to international guidelines.[4]

Alzheimer Dementia Cohort

For the Alzheimer's disease cohort, patients underwent a standardized dementia assessment including medical history, informant-based history, physical and neurologic examination, laboratory tests, neuropsychological testing, EEG, and MRI of the brain. Patients with the diagnosis of Alzheimer's disease, made according to clinical diagnostic criteria, were included in this study.

p3. Healthy control cohort

Controls from the Amsterdam Dementia Cohort were individuals who were referred for cognitive complaints between November 2005 and July 2023, in whom all clinical investigations were normal (i.e. criteria for MCI or any psychiatric or neurological disorder not fulfilled). These individuals were labelled as having subjective cognitive decline and were included as controls. For the Progress-PD, CSF of healthy controls was collected at the outpatient movement disorders clinic of Amsterdam UMC, location VUmc, in period 2008-2010 and 2017-2018, and stored at −80 °C in the Amsterdam UMC biobank until further processing.

For the healthy plasma control cohort, we used plasma samples of healthy individuals included in the MeninGene Recall study.[5] Details of this study have been published previously. In short, the healthy individuals were partners or proxies of meningitis patients who were included in the MeninGene study between October 2011 and March 2015. One to five years after admission, patients and healthy controls were recalled to the Amsterdam UMC for blood withdrawal.[5]

p4. Reagents list

Company	Description	Category number	RRID (if available)
Magnetic COOH beads			
BioRad	Magnetic COOH beads, region 26	MC1-0026-01	
BioRad	Magnetic COOH beads, region 27	MC1-0027-01	
BioRad	Magnetic COOH beads, region 52	MC1-0028-01	
BioRad	Magnetic COOH beads, region 29	MC1-0029-01	
BioRad	Magnetic COOH beads, region 34	MC1-0034-01	
BioRad	Magnetic COOH beads, region 35	MC1-0035-01	
BioRad	Magnetic COOH beads, region 36	MC1-0036-01	
BioRad	Magnetic COOH beads, region 43	MC1-0043-01	
BioRad	Magnetic COOH beads, region 45	MC1-0045-01	
BioRad	Magnetic COOH beads, region 53	MC1-0053-01	
BioRad	Magnetic COOH beads, region 54	MC1-0054-01	
BioRad	Magnetic COOH beads, region 65	MC1-0065-01	
BioRad	Magnetic COOH beads, region 63	MC1-0063-01	
BioRad	Magnetic COOH beads, region 37	MC1-0037-01	
BioRad	Magnetic COOH beads, region 44	MC1-0063-02	
BioRad	Magnetic COOH beads, region 46	MC1-0037-02	
Recombinant proteins (CF = carrier-free)			
R&D	rh IFNg, CF	285-IF-100/CF	
R&D	rh IL-17A, CF	7955-IL-025/CF	
R&D	rh TNFa, CF	10291-TA-050	
Peprotech	rh GM-CSF	300-03	
R&D	rh IFNa, CF	11101-2	
Peprotech	rh IL-12p70	200-12	
R&D	rh IL-6, CF	7270-IL-025/CF	
Peprotech	rh IFNb	300-02BC	
R&D	rh IL-1a, CF	200-LA-010/CF	
R&D	rh IFNL2, CF	8417-IL-025/CF	
R&D	rh IFNL3, CF	5259-IL-025/CF	
Peprotech	rh IL-17F	200-25	
Peprotech	rh IL-23	200-23	
Peprotech	rh IFNw	300-02J	
R&D	rh TGF-beta 1	7754-BH-025/CF	
R&D	rh IFNL1, CF	1598-IL-025/CF	
Commercial antibodies (positive controls)			
R&D	anti-IFNg	MAB285-SP	AB_2123306
ThermoFisher	anti-IL-17A	16-7178-85	AB_494122
ThermoFisher	anti-TNFa	14-7348-85	AB_469259
R&D	anti-GM-CSF	MAB215-SP	AB_2229972
BD biosciences	anti-IFNa	551795	AB_394252
R&D	anti-IL-12p70	MAB611R-SP	AB_3658538
R&D	anti-IL-6	MAB206-SP	AB_2127617

R&D	anti-IFNb	21400-1	AB_354169
R&D	anti-IL-1a	MAB200-SP	AB_2295862
R&D	anti-IFNL2	MAB1587-SP	AB_2125211
R&D	anti-IFNL3	MAB15981-SP	AB_2125340
R&D	anti-IL-17F	MAB13352-SP	AB_3657622
R&D	anti IL-23	MAB17161	AB_10973762
ThermoFisher	anti-IFNw	BMS1021	AB_10596364
R&D	anti-TGFb	MAB2401-SP	AB_358120
R&D	anti-human IFNL1	MAB15981-SP	AB_2125340
Bead assay reagents			
ThermoFisher	anti-human IgG (PE) secondary	12-4998-82	AB_465926
ThermoFisher	anti-mouse IgG (PE) secondary	12-4010-87	AB_11044909

p5. Competitive binding assay

To confirm the specificity of the autoantibodies targeting IFN-ω and IL-17A, a competitive binding assay was performed. Samples were centrifuged, subsequently the appropriate concentration of cytokine or PBS was added to the sample followed by 60 minutes of incubation at 37 °C. Subsequently, the protocol for autoantibody detection as described above was followed. The presence of excess cytokine reduced reactivity against IFN-ω- and IL-17A-coupled beads, confirming that the autoantibodies specifically bind to the cytokines rather than the beads (appendix p10).

p6. Functional evaluation of anti-cytokine autoantibodies

STAT phosphorylation

The impact of autoantibody-containing CSF versus that of autoantibody-negative CSF on cytokine-specific signal transduction in isolated normal peripheral blood mononuclear cells (PBMCs) was assessed by flow cytometry. Assay details are outlined in appendix p12 and p18. All cells were fixed and stained for known intracellular phosphorylated products like phosphorylated signal transducers and activator of transcription (pSTAT) downstream of the ligand-receptor pair as described before.[6] Data were collected using FACSSymphony A1 (BD Biosciences), analysed using FACS Diva Software and FlowJo (Treestar, v10·9.0).

HEK-Blue cells

HEK-Blue cells (InvivoGen) are HEK 293 cells engineered to detect their target cytokine and produce SEAP in response. Assays were performed according to manufacturer's protocols with assay details and representative

results shown in appendix p12 and 18, respectively. In short, cells were cultured in DMEM with appropriate antibiotics and 10% of patient or normal plasma or CSF. Cells were stimulated overnight with cytokine and the next day SEAP production was quantified using the QUANTI-Blue (Invivogen) colorimetric enzyme assay.

p7. Bacterial load quantification and cytokine measurements in CSF

To determine bacterial load, 200 µL of CSF was centrifuged (10 minutes, 4500 x g) and after treatment with a lysozyme/lysostaphin digestion buffer DNA was extracted using the QIAamp® DNA Mini Kit (QIAGEN, Hilden, Germany). Real-time PCRs were performed using the CFX96 Real-time PCR System (BIO-RAD, the Netherlands). Quantification of the *S. pneumoniae* bacterial load was performed using a primer targeting the autolysin gene (*lytA*), which has been shown to be specific for *S. pneumoniae* and provides accurate quantification.[7] External standard curves were created using genomic DNA extracted from *S. pneumoniae* ATCC 6303.

SUPPLEMENTARY FIGURES

p8. Supplementary Figure 1. Heatmap with positive autoantibody in CSF of pneumococcal meningitis patients. Heatmap showing the clustering of positive autoantibodies in the CSF of pneumococcal meningitis patients with at least 1 positive autoantibody (282 of 623 [45%]). Heatmap was made using "ComplexHeatmap" package in Rstudio, with Ward.D2 clustering method.

p9. Supplementary Figure 2. Cytokine measurements in the CSF of pneumococcal meningitis patients. a-b, Cytokine levels in patients with autoantibodies against IFN-ω (N = 41) compared to those without (N = 361) **(a)** and in patients with antibodies against IL-17A (N = 41) or those without (N = 361) **(b)**. Cytokines were measured with Luminex technology. Ns, not significant.

p10. Supplementary Figure 3. Autoantibody measurements in the plasma of pneumococcal meningitis patients. a, Correlation of autoantibody levels against IFN-ω and IL-17A in the plasma and CSF. Autoantibodies are measured in 100x diluted plasma and undiluted CSF. Shown are patients positive for autoantibody in both compartments (green dots), negative in both compartments (black dots), positive only in CSF (red dots) and positive only in the plasma (blue dots). **b,** Multiplex particle-based assay for autoantibodies against GM-CSF,

IFN-α, IFN-β, IFN-γ, IFN-λ2, IFN-λ3, IFN-ω, IL-1α, IL-6, IL-12, IL-17A, IL-17F, IL-23, TGF-β in the plasma of patients with pneumococcal meningitis in the acute phase (day 0-2 after presentation; N = 83), subacute phase (day 7; N = 58) or late phase (day 90; N =35) after presentation. **c,** Autoantibodies against IFN-ω and IL-17A over time. Measurements in the same patients are connected. The red dotted line shows the cut-off for positivity. CSF, cerebrospinal fluid.

p11. Supplementary Figure 4. Polyreactivity. Specificity of autoantibodies against IFN-ω and IL-17A confirmed using a competitive binding assay. Reactivity against IFN-ω and IL-17A coupled beads decreased in the presence of excess cytokine, showing that the autoantibodies specifically bind to the cytokine.

p12. Supplementary Figure 5. Pilot anticytokine autoantibody measurements. a-b, Multiplex particle-based assay for autoantibodies against C3a, C5a, CXCL10, GM-CSF, IFN-α, IFN-β, IFN-γ, IFN-λ1, IFN-λ2, IFN-λ3, IFN-ω, IL-1α, IL-6, IL-10, IL-12, IL-15, IL-17A, IL-17F, IL-22, IL-23, IL-33, M-CSF, TGF-β, TNF and TNF-β in the CSF of patients with pneumococcal **(a)** and healthy controls **(b)**.

p13. Supplementary Figure 6. Representative neutralisation assays. a, Representative FACS plots depicting IFN-γ (upper panel) and IFN-ω (lower panel) induced pSTAT1 levels in healthy donor cells (gated on CD14+ monocytes) in the presence of healthy control CSF, PBS with anti-IFN-γ or anti-IFN-ω commercial antibody, or anti-IFN-γ or anti-IFN-ω positive patient CSF. **b,** Representative results of cytokine induced secreted embryonic alkaline phosphatase (SEAP) measured by colorimetric assay in IL-17, IL-1α and IL-6 reporter HEK 293 cells (HEK-Blue cells) in the presence of healthy control CSF, PBS with appropriate commercial antibody, or in antibody positive patient CSF. Assays measuring neutralisation of anti-IL-1α and anti- IL-6 antibodies were unsuccessful due to patient CSF stimulating the cells in the absence of cytokine.

p8. Supplementary Figure 1

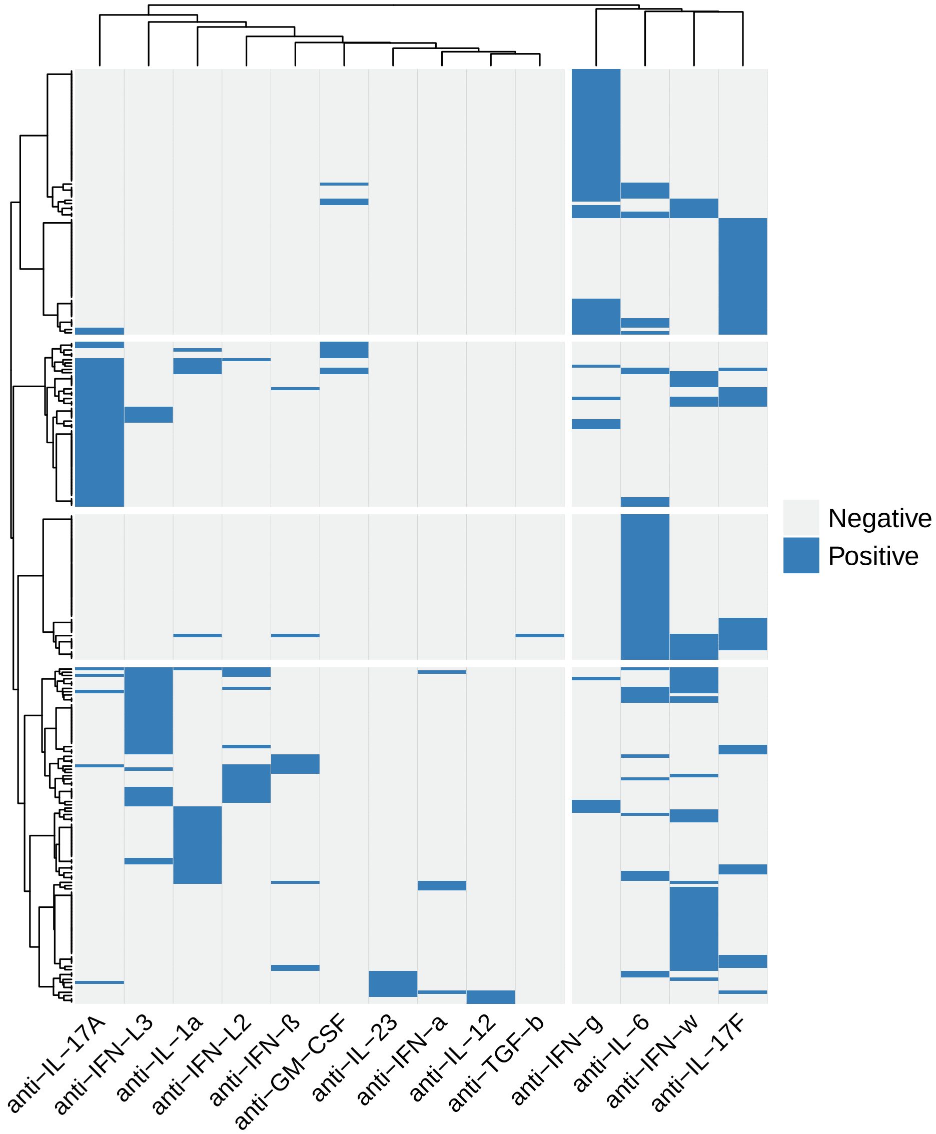

p9. Supplementary Figure 2

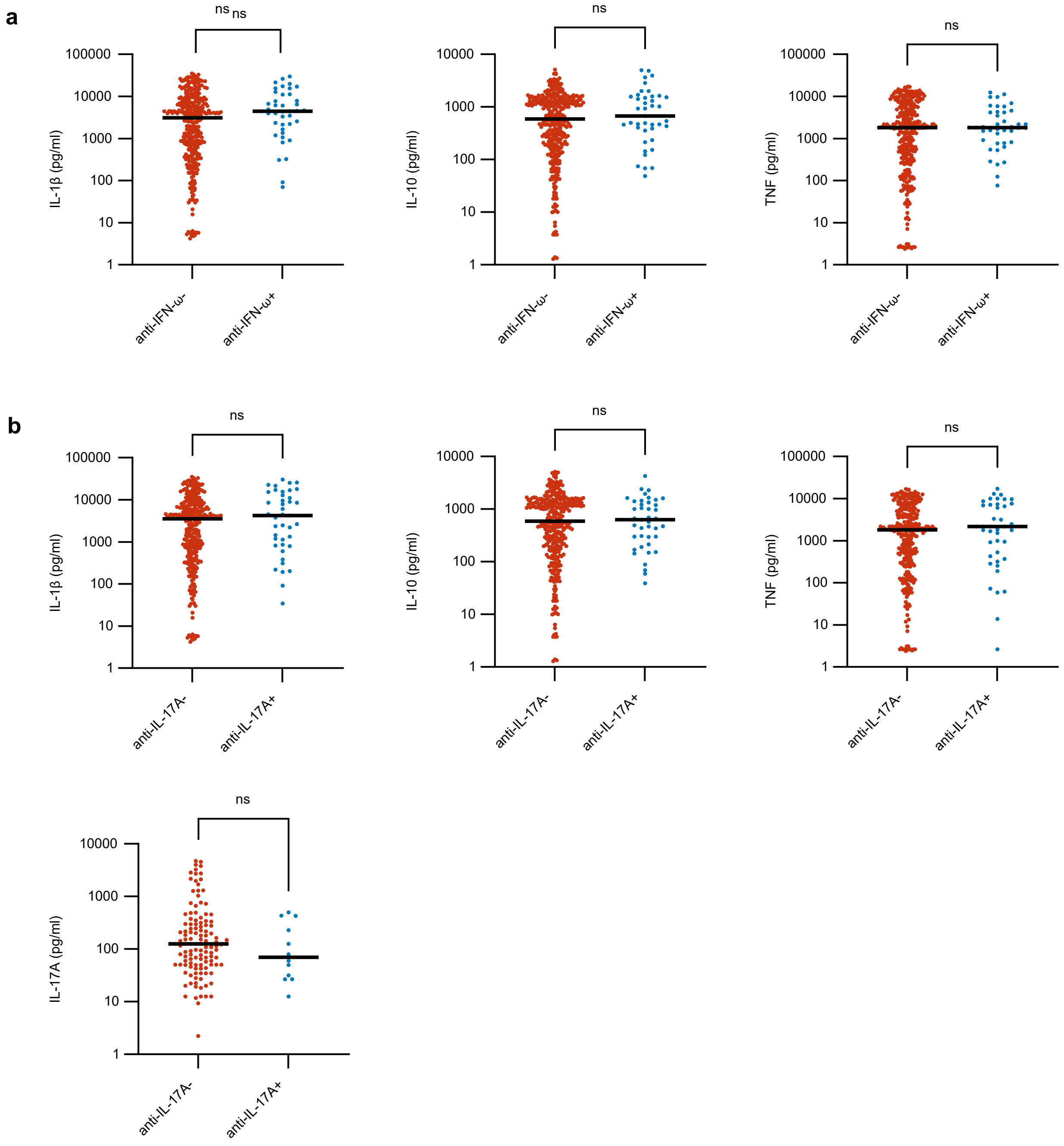

p10. Supplementary Figure 3

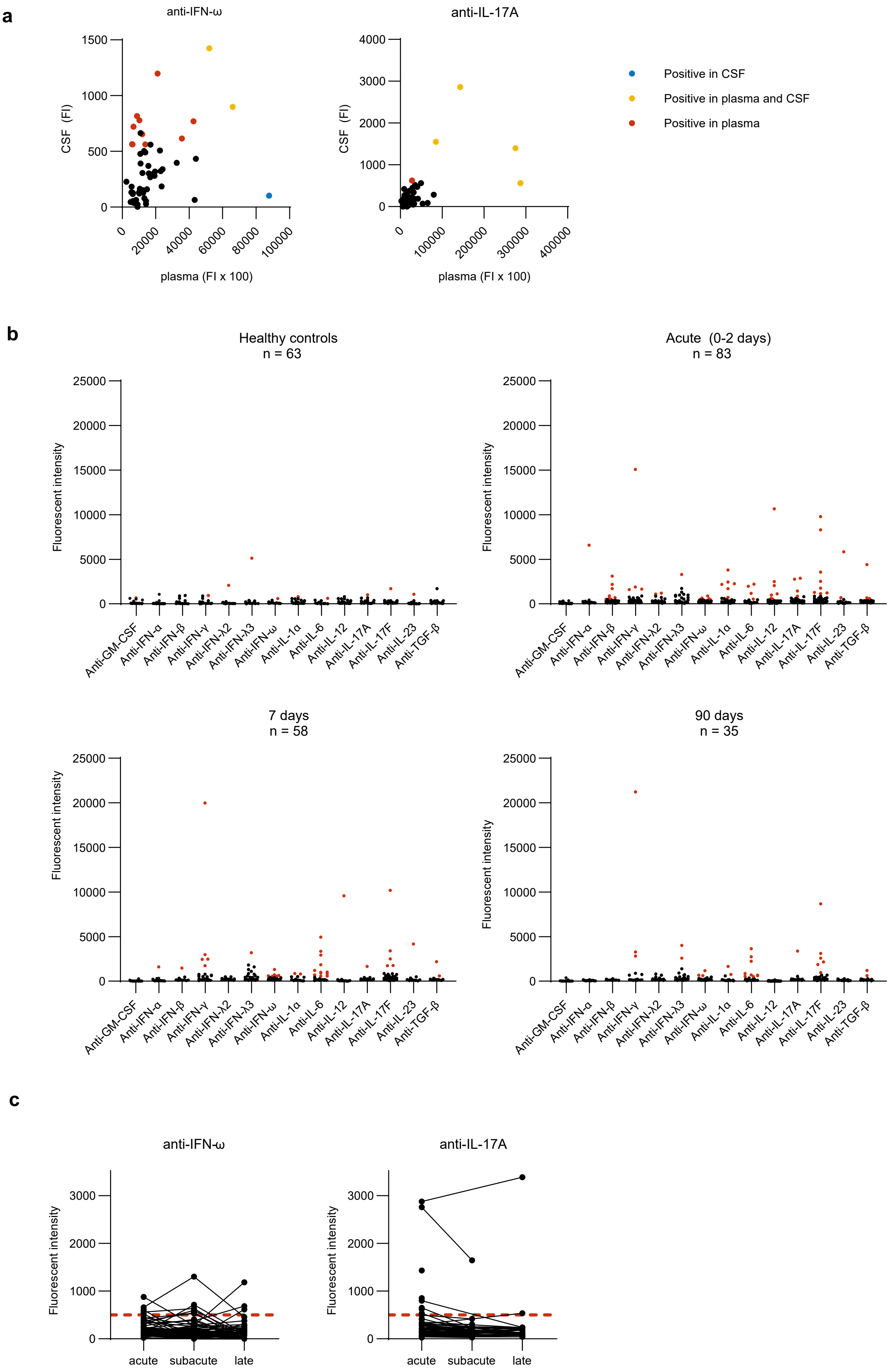

p11. Supplementary Figure 4

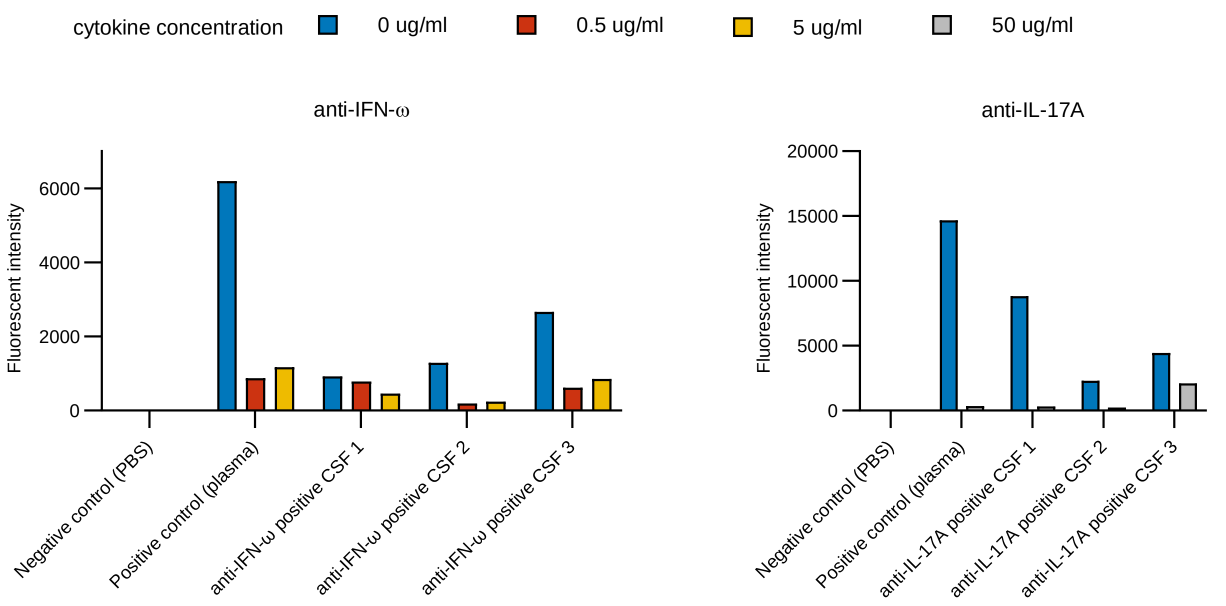

p12. Supplementary Figure 5

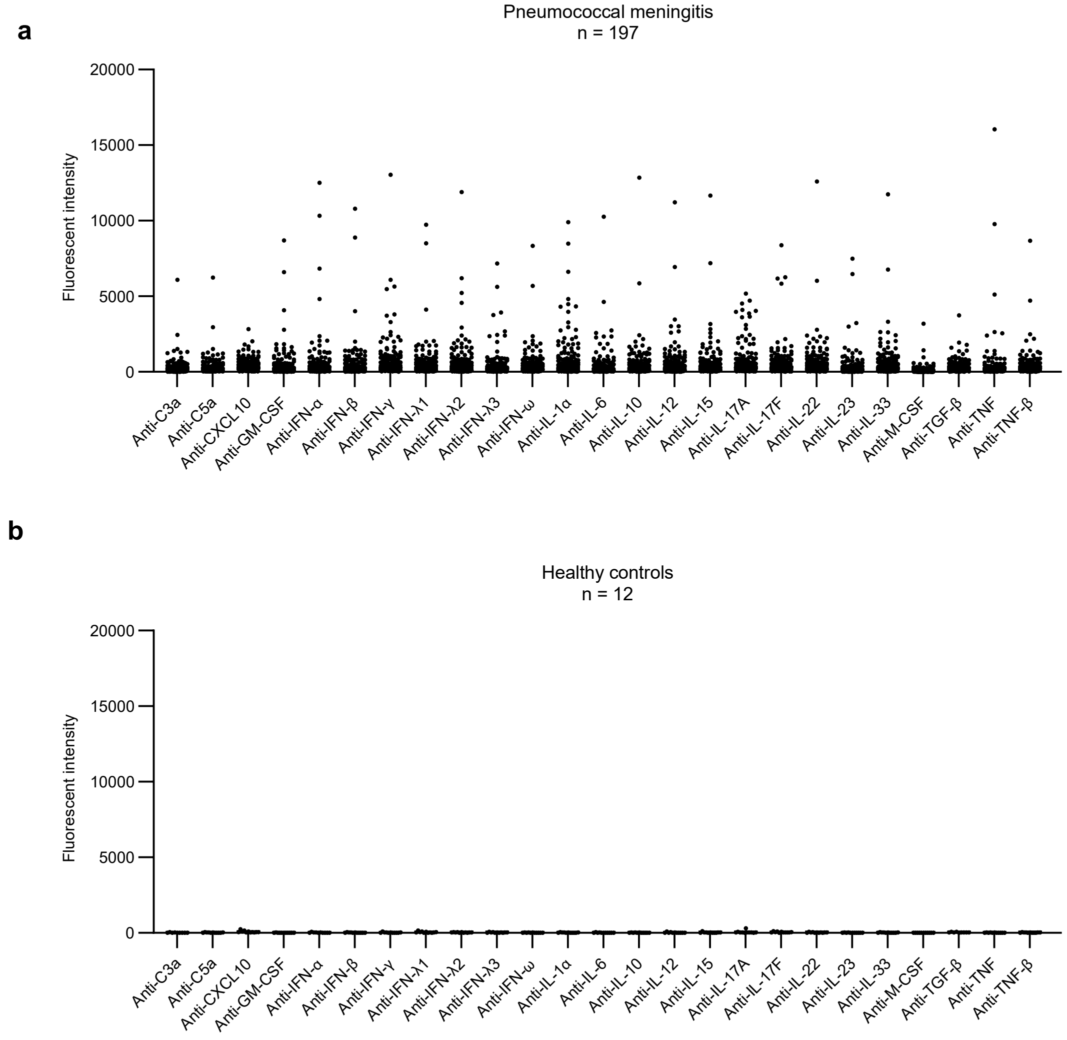

p13. Supplementary Figure 6

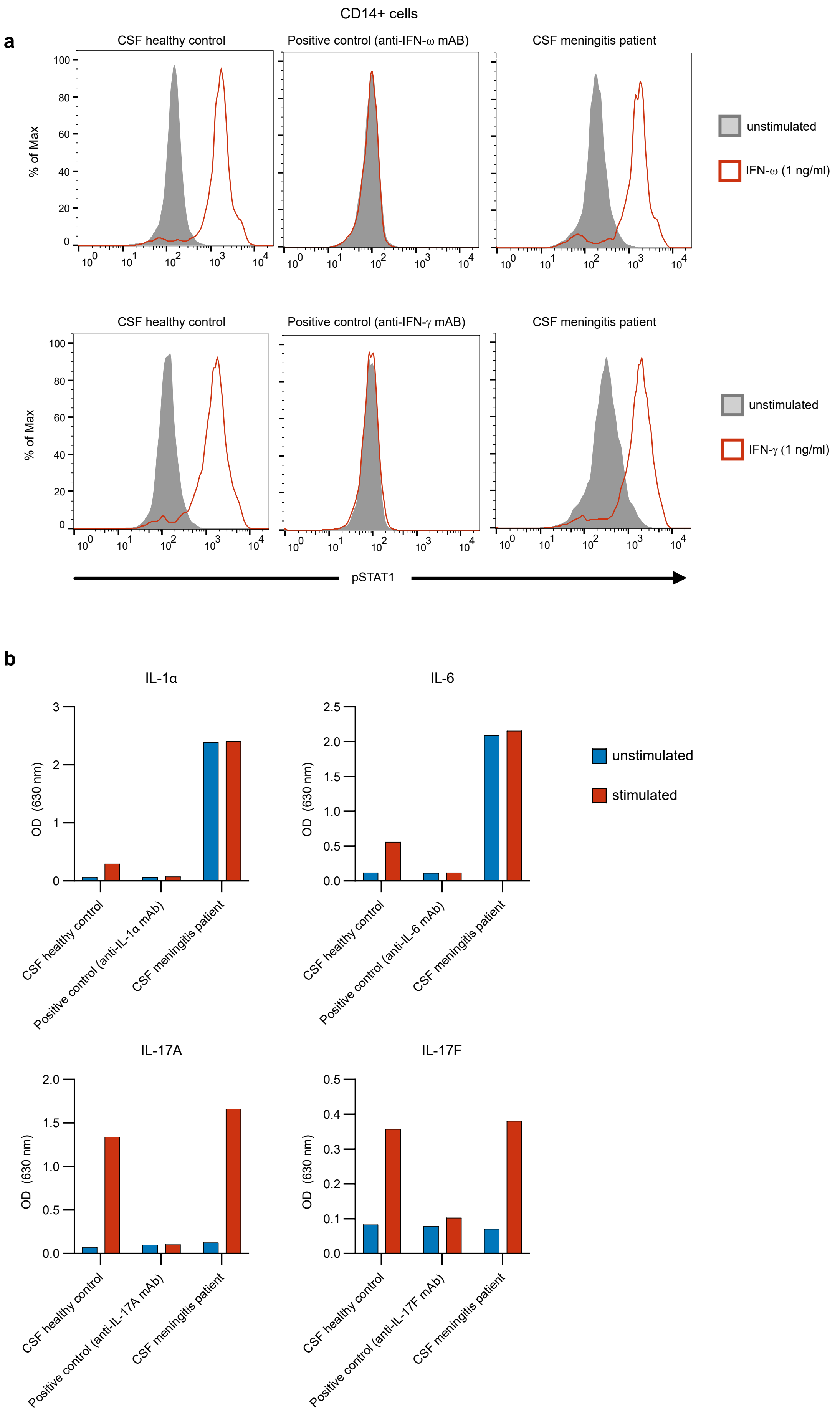

SUPPLEMENTARY TABLES

p14. Supplementary Table 1. Baseline and clinical characteristics comparing pneumococcal and meningococcal meningitis patients included in the MeninGene study with and without cerebrospinal fluid available.

Characteristic	Pneumococcal meningitis patients			Meningococcal meningitis patients		
	**No CSF available n = 1,320**	**CSF available n = 623**	**p-value**	**No CSF available n = 216**	**CSF available n = 86**	**p-value**
Age - years[a]	62 (52-70)	62 (51-70)	0.64	31 (19 – 54)	22 (18 – 53)	0.33
Female sex	667/1,320 (51%)	307/623 (49%)	0.63	111/216 (51%)	45/86 (52%)	0.9
Recurrent meningitis	80/1,318 (6%)	40/622 (6%)	0.76	9/216 (4%)	2/86 (2%)	0.73
Immunocompromised	393/1,319 (30%)	200/623 (32%)	0.32	32/216 (15%)	8/86 (9%)	0.26
**Clinical signs and symptoms on admission**	-	-	-	-	-	-
Temperature (°C)[b]	38.9 (37.9-39.6)	39.0 (38.0-39.7)	0.17	38.1 (37.2-39.0)	38.0 (36.9-39.0)	0.90
Altered mental status (GCS <14)	1,027/1,313 (78%)	498/622 (80%)	0.37	109/214 (51%)	33/86 (38%)	0.055
Classic triad[c]	540/1,221 (44%)	268/581 (46%)	0.48	45/216 (21%)	15/85 (17%)	0.63
Seizures	132/1,254 (11%)	61/598 (10%)	0.87	7/210 (3%)	3/82 (4%)	>0.99
**Indices of CSF inflammation**	-	-	-	-	-	-
Leukocytes - cells/mm3[d]	2,237 (478-6,430)	2,755 (644-6,667)	0.077	6,260 (1,793 – 12,302)	5,243 (2,049 – 12,272)	0.4
<100/mm^3	150 (14%)	65 (13%)	-	10 (7.1%)	8 (14%)	-
100–999/mm^3	296 (27%)	118 (23%)	-	13 (9.2%)	7 (12%)	-
>999/mm^3	642 (59%)	329 (64%)	-	118 (84%)	43 (74%)	-
**Glasgow Outcome Scale**	-	-	0.11	-	-	0.39
1 (death)	251/1,320 (19%)	91/623 (15%)	-	9/216 (4%)	1/86 (1%)	-
2 (vegetative state)	1/1,320 (0%)	1/623 (0%)	-	0/216 (0%)	0/86 (0%)	-
3 (severe disability)	65/1,320 (5%)	31/623 (5%)	-	4/216 (2%)	1/86 (1%)	-

Characteristic	Pneumococcal meningitis patients			Meningococcal meningitis patients		
	**No CSF available**<br>**n = 1,320**	**CSF available**<br>**n = 623**	**p-value**	**No CSF available**<br>**n = 216**	**CSF available**<br>**n = 86**	**p-value**
4 (moderate disability)	246/1,320 (19%)	113/623 (18%)	-	18/216 (8%)	11/86 (13%)	-
5 (mild or no disability)	757/1,320 (57%)	387/623 (62%)	-	185/216 (86%)	73/86 (85%)	-

Data presented as n/N (%) or median (IQR). Group differences were tested with a Fisher's exact test for categorical variables and a Mann–Whitney U test for continuous variables. Abbreviations: CSF, cerebrospinal fluid. [a]Age is known for all patients. [b]Temperature is known for 1680 pneumococcal meningitis episodes, and 294 meningococcal meningitis episodes. [c]Classic triad is defined as the combination of headache, neck stiffness and altered mental status. [d]Leukocyte count in CSF is known for 1903 pneumococcal meningitis episodes, and 292 meningococcal meningitis episodes.

p15. Supplementary Table 2. Baseline and clinical characteristics of plasma cohort *Streptococcus pneumoniae* meningitis (n=83)

Characteristic	Data	Characteristic	Data
Age, years[a]	64 (55-70)	White cell count (per mm^3)[k]	2,910 (830-5,469)
Female sex	29/83 (35%)	<100 ⁄mm^3	6/83 (8.7%)
Symptoms <24 hours	43/80 (54%)	100–999 ⁄mm^3	16/83 (24%)
Recurrent meningitis	4/83 (5%)	>999 ⁄mm^3	46/83 (68%)
Extrameningeal focus of infection	45/83 (54%)	**Microbiology blood and CSF**	
Antibiotics before admission	10/83 (12%)	Positive CSF culture	60/83 (72%)
Immunocompromised state[b]	29/83 (35%)	Positive blood culture	63/83 (75%)
**Clinical signs and symptoms on admission**		Only positive CSF PCR	5/83 (6%)
Median temperature[c]	38.8 (38-39.5)	**Clinical course**	
Fever (>38 ºC)	61/81 (75%)	Cardiorespiratory failure	30/82 (36%)
Headache	51/65 (78%)	Sinus thrombosis	2/73 (3%)
Nausea	33/65 (51%)	Cerebrovascular accident	14/77 (18%)
Neck stiffness	58/77 (75%)	Seizures	17/78 (22%)
Glasgow Coma Scale Score[d]	10 (8-13)	Hydrocephalus	7/76 (9%)
Altered mental status (GCS <14)	65/83 (78%)	**Glasgow Outcome Score**	
Coma (GCS ≤8)	25/83 (30%)	1 (death)	20/83 (24%)
Aphasia, monoparesis or hemiparesis	22/75 (29%)	2 (vegetative state)	1/83 (1%)
Seizures	14/82 (17%)	3 (severe disability)	5/83 (6%)
Cranial nerve palsies	11/77 (14%)	4 (moderate disability)	12/83 (14%)
Heart rate (beats/min)[e]	96 (81-113)	5 (mild or no disability)	45/83 (55%)
**Blood chemical tests**		**Measured blood and CSF samples**	
C-reactive protein (mg/L)[f]	179 (68-280)	CSF	56/83 (67%)
Thrombocyte count (per µL)[g]	199 (145-255)	Blood, day 0	45/83 (54%)
Leukocyte count (per µL)[h]	17.1 (10.8-22.8)	Blood, day 1	29/83 (35%)
**Indices of CSF inflammation**		Blood, day 2	9/83 (11%)
Protein (g ⁄ L)[i]	4.84 (2.54-6.78)	Blood, day 7	58/83 (70%)
CSF ⁄ serum glucose ratio[j]	0.07 (0.01-0.28)	Blood, day 90	35/83 (42%)

Data presented as n/N (%) or median (IQR). Abbreviations: CSF, cerebrospinal fluid; PCR, polymerase chain reaction. [a]Age is known for all patients. [b]Immunocompromised state is defined as active cancer (N = 7), diabetes (N = 14), alcoholism (N = 9), immunosuppressive treatment (N = 5), splenectomy (N = 0) or HIV (N =1). [c]Temperature is known for 81 episodes. [d]Glasgow Coma Scale score is known for all episodes. [e]Heart rate is known for 81 episodes. [f]C-reactive protein in blood is known for all episodes. [g]Thrombocyte count in blood is known for 81 episodes. [h]Leukocyte count in blood is known for all episodes. [i]Protein level in CSF is known for 78 episodes. [j]Glucose ratio is known for 79 episodes. [k]White cell count in CSF is known for 78 episodes.

p16. Supplementary Table 3. Baseline and clinical characteristics of cerebrospinal fluid cohort of *Neisseria meningitidis* meningitis (n=86)

Characteristic	Data	Characteristic	Data
Age, years[a]	22 (18-53)	**Indices of CSF inflammation**	
Female sex	45/86 (52%)	Opening pressure (cm H2O)[k]	45 (33-50)
Symptoms <24 hours	35/82 (43%)	Protein (g ∕ L)[l]	4.00 (1.69-6.02)
Recurrent meningitis	2/86 (2%)	CSF ∕ serum glucose ratio[m]	0.06 (0.01-0.28)
Extrameningeal focus of infection	5/86 (6%)	White cell count (per mm^3)[n]	5,243 (2,130-11,700)
Otitis or sinusitis	4/83 (5%)	<100 ∕ mm^3	8 (14%)
Pneumonia	1/86 (1%)	100–999 ∕ mm^3	7 (12%)
Immunocompromised state[b]	8/86 (9%)	>999 ∕ mm^3	43 (74%)
**Clinical signs and symptoms on admission**		**Microbiology blood and CSF**	
Median temperature[c]	38.0 (36.9-39.0)	Positive CSF culture	68/86 (79%)
Fever (>38 ºC)	42/83 (51%)	Positive blood culture	30/55 (55%)
Headache	73/80 (91%)	Only positive CSF PCR	17/86 (20%)
Nausea	62/76 (82%)	**Clinical course**	
Neck stiffness	67/81 (83%)	ICU admission	33/86 (38%)
Glasgow Coma Scale Score[d]	14 (11-15)	Cardiorespiratory failure	12/83 (12%)
Altered mental status (GCS <14)	33/86 (38%)	Mechanical ventilation	12/86 (14%)
Coma (GCS ≤8)	7/86 (8%)	Cerebrovascular accident	1/83 (1%)
Classic triad[e]	15/85 (18%)	Seizures	1/83 (1%)
Aphasia, monoparesis or hemiparesis	7/83 (8%)	Hydrocephalus	28/583 (5%)
Seizures	3/82 (4%)	**Glasgow Outcome Score**	
Cranial nerve palsies	3/75 (4%)	1 (death)	1/86 (1%)
Heart rate (beats/min)[f]	91 (76-106)	2 (vegetative state)	0/86 (0%)
Systolic blood pressure (mmHg)[g]	129 (115-141)	3 (severe disability)	1/86 (1%)
**Blood chemical tests**		4 (moderate disability)	11/86 (13%)
C-reactive protein (mg/L)[h]	230 (132-296)	5 (mild or no disability)	73/86 (85%)
Thrombocyte count (per µL)[i]	197 (158-253)	**Neurological sequelae at discharge**	
Leukocyte count (per µL)[j]	19.4 (14.1-24.1)	Cognitive impairment	13/77 (17%)
		Cranial nerve palsy	3/55 (5%)

Data presented as n/N (%) or median (IQR). Abbreviations: CSF, cerebrospinal fluid; PCR, polymerase chain reaction; ICU, intensive care unit. [a]Age is known for all patients. [b]Immunocompromised state is defined as active cancer (n = 3), diabetes (n = 4), alcoholism (n = 1), immunosuppressive treatment (n = 1), splenectomy (N = 0) or HIV (N =0). [c]Temperature is known for 83 episodes. [d]Glasgow Coma Scale score is known for all episodes. [e]Classic triad is defined as the combination of headache, neck stiffness and altered mental status. [f]Heart rate is known for 84 episodes. [g]Systolic blood pressure is known for 82 episodes. [h]C-reactive protein in blood is known for 83 episodes. [i]Thrombocyte count in blood is known for 83 episodes. [j]Leukocyte count in blood is known for all episodes. [k]Opening pressure is known for 47 episodes. [l]Protein level in CSF is known for 84 episodes. [m]Glucose ratio is known for 82 episodes. [n]White cell count in CSF is known for 85 episodes.

p17. Supplementary Table 4. Baseline characteristics of non-bacterial meningitis cohorts

Characteristic	Data	Characteristic	Data
**Viral meningitis/ encephalitis CSF cohort (N = 56)**		**Alzheimer's disease CSF cohort (N = 206)**	
Age, years[a]	43 (30 – 58)	Age, years[a]	65 (62 – 68)
Female sex	35/56 (63%)	Female sex	109/206 (53%)
Final diagnosis		**Parkinson's disease CSF cohort (N = 61)**	
Varicella zoster meningitis	9/56 (16%)	Age, years[a]	64 (59 – 69)
Varicella zoster encephalitis	7/56 (13%)	Female sex	20/61 (33%)
Herpes simplex virus type 1/2 meningitis	9/56 (16%)	**Healthy individuals CSF cohort (N = 264)**	
Herpes simplex virus type 1/2 encephalitis	13/56 (23%)	Age, years[a]	62 (59 – 66)
Tick-borne encephalitis	1/56 (2%)	Female sex	107/264 (41%)
HIV meningitis	2/56 (4%)	**Healthy individuals plasma cohort (N = 63)**	
Enterovirus meningitis	12/56 (21%)	Age, years[a]	65 (55 – 69)
Human herpes virus type 6 encephalitis	2/56 (4%)	Female sex	30/63 (48%)
Epstein-Barr-virus encephalitis	1/56 (2%)		

Data presented as n/N (%) or median (IQR). Abbreviations: CSF, cerebrospinal fluid; PCR, polymerase chain reaction; HIV, human immunodeficiency virus

p18. Supplementary Table 5. Neutralising capacity of cerebrospinal fluid or blood of bacterial meningitis patients

Antibody	Positive patients	Tested	Neutralising
**Pneumococcal meningitis (cerebrospinal fluid)**			
Anti-IFN-γ	66/622 (11%)	26/66 (39%)	0
Anti-IFN-ω	66/624 (11%)	12/66 (18%)	1/12 (8%)
Anti-IL-17A	55/624 (9%)	23/55 (42%)	0
Anti-IL-17F	64/624 (10%)	14/64 (22%)	0
**Meningococcal meningitis (cerebrospinal fluid)**			
Anti-IFN-γ	9/86 (11%)	9/86 (11%)	1/6 (17%)
Anti-IFN-ω	1/86 (1%)	1/86 (1%)	0
Anti-IL-17A	3/86 (3%)	3/86 (3%)	0
Anti-IL-17F	6/86 (7%)	6/86 (7%)	0
**Pneumococcal meningitis (plasma or serum)**			
Anti-IFN-γ	6/83 (7%)	7/9 (78%)	0
Anti-IFN-ω	10/83 (12%)	1/1 (100%)	0
Anti-IL-17A	4/83 (5%)	0/3 (0%)	na
Anti-IL-17F	9/83 (11%)	0/6 (0%)	na

Data presented as n/N (%). Neutralisation capacity of undiluted CSF or 1/10 diluted plasma was tested at a concentration of 1 ng/ml of IFN-ω or IFN-γ. Neutralisation capacity of 1/10 diluted CSF or serum was tested at a concentration of 1 or 3 ng/ml of IL-17A and IL-17F. For plasma or serum samples the time point with the highest level of autoantibody was tested.

p19. Supplementary Table 6. Functional assays to assess biological activity of anticytokine autoantibodies *in vitro.*

Cytokine (conc)	Incubation (time)	Signalling target	Cell type	Method	Sample
IFN-γ (1 ng/ml)	15 min	pSTAT1	CD14+ monocytes	Flow, surface and IC staining	1/10 plasma in RPMI medium or undiluted CSF
IFN-ω (1 ng/ml)	15 min	pSTAT1	CD14+ monocytes	Flow, surface and IC staining	1/10 plasma in RPMI medium or undiluted CSF
IL-1α	24 hours	NF-kB and AP-1	HEK-Blue IL-1R cells	Colorimetric assay	1/10 plasma or CSF in DMEM
IL-6	24 hours	STAT3	HEK-Blue IL-6 cells	Colorimetric assay	1/10 plasma or CSF in DMEM
IL-12 (25 ng/ml)	15 min	pSTAT4	CD4+ T cells activated with anti-CD3 and anti-CD28	Flow, surface and IC staining	1/10 plasma in RPMI medium or undiluted CSF
IL-17A (1 ng/ml)	24 hours	NF-kB and AP-1	HEK-Blue IL-17 cells	Colorimetric assay	1/10 plasma or CSF in DMEM
IL-17F (3 ng/ml)	24 hours	NF-kB and AP-1	HEK-Blue IL-17 cells	Colorimetric assay	1/10 plasma or CSF in DMEM

Abbreviations: STAT, signal transducer and activator of transcription; NF-kB, Nuclear factor kappa-light-chain-enhancer of activated B-cells; CD, cluster of differentiation; RPMI, Roswell Park Memorial Institute; DMEM, Dulbecco's Modified Eagle Medium.

SUPPLEMENTARY REFERENCES

1. Olie SE, Staal SL, Ter Horst L, et al. Diagnostic accuracy of inflammatory markers in adults with suspected central nervous system infections. *J Infect* 2024; **88**(3): 106117.
2. Postuma RB, Berg D, Stern M, et al. MDS clinical diagnostic criteria for Parkinson's disease. *Mov Disord* 2015; **30**(12): 1591-601.
3. Hughes AJ, Daniel SE, Kilford L, Lees AJ. Accuracy of clinical diagnosis of idiopathic Parkinson's disease: a clinico-pathological study of 100 cases. *J Neurol Neurosurg Psychiatry* 1992; **55**(3): 181-4.
4. Teunissen CE, Petzold A, Bennett JL, et al. A consensus protocol for the standardization of cerebrospinal fluid collection and biobanking. *Neurology* 2009; **73**(22): 1914-22.
5. Kloek AT, Seron MV, Schmand B, et al. Individual responsiveness of macrophage migration inhibitory factor predicts long-term cognitive impairment after bacterial meningitis. *Acta Neuropathologica Communications* 2021; **9**(1): 4.
6. Gupta S, Tatouli IP, Rosen LB, et al. Distinct Functions of Autoantibodies Against Interferon in Systemic Lupus Erythematosus: A Comprehensive Analysis of Anticytokine Autoantibodies in Common Rheumatic Diseases. *Arthritis Rheumatol* 2016; **68**(7): 1677-87.
7. Ganaie FA, Govindan V, Ravi Kumar KL. Standardisation and evaluation of a quantitative multiplex real-time PCR assay for the rapid identification of Streptococcus pneumoniae. *Pneumonia (Nathan)* 2015; **6**: 57-66.
